# Monkey Pulvinar Neurons Fire Differentially to Snake Postures

**DOI:** 10.1371/journal.pone.0114258

**Published:** 2014-12-05

**Authors:** Quan Van Le, Lynne A. Isbell, Jumpei Matsumoto, Van Quang Le, Etsuro Hori, Anh Hai Tran, Rafael S. Maior, Carlos Tomaz, Taketoshi Ono, Hisao Nishijo

**Affiliations:** 1 System Emotional Science, Graduate School of Medicine and Pharmaceutical Sciences, University of Toyama, Toyama, Japan; 2 Department of Anthropology, University of California Davis, Davis, California, 95616, United States of America; 3 Primate Center and Laboratory of Neurosciences and Behavior, Department of Physiological Sciences, Institute of Biology, University of Brasília, Brasilia, DF, Brazil; University of Reading, United Kingdom

## Abstract

There is growing evidence from both behavioral and neurophysiological approaches that primates are able to rapidly discriminate visually between snakes and innocuous stimuli. Recent behavioral evidence suggests that primates are also able to discriminate the level of threat posed by snakes, by responding more intensely to a snake model poised to strike than to snake models in coiled or sinusoidal postures (Etting and Isbell 2014). In the present study, we examine the potential for an underlying neurological basis for this ability. Previous research indicated that the pulvinar is highly sensitive to snake images. We thus recorded pulvinar neurons in Japanese macaques (*Macaca fuscata*) while they viewed photos of snakes in striking and non-striking postures in a delayed non-matching to sample (DNMS) task. Of 821 neurons recorded, 78 visually responsive neurons were tested with the all snake images. We found that pulvinar neurons in the medial and dorsolateral pulvinar responded more strongly to snakes in threat displays poised to strike than snakes in non-threat-displaying postures with no significant difference in response latencies. A multidimensional scaling analysis of the 78 visually responsive neurons indicated that threat-displaying and non-threat-displaying snakes were separated into two different clusters in the first epoch of 50 ms after stimulus onset, suggesting bottom-up visual information processing. These results indicate that pulvinar neurons in primates discriminate between poised to strike from those in non-threat-displaying postures. This neuronal ability likely facilitates behavioral discrimination and has clear adaptive value. Our results are thus consistent with the Snake Detection Theory, which posits that snakes were instrumental in the evolution of primate visual systems.

## Introduction

Biologically threatening stimuli are known to elicit fast and vigorous reactions from most animals, including freezing, darting, rapid retreat, alarm calls, and defensive attacks. Such responses were likely selected for over evolutionary time because animals that behaved in ways that helped them avoid threatening stimuli would have survived longer than those that did not. Before these reactions occur, however, the threatening stimuli must be detected, and this can be achieved through one or more of the senses. Rodents for example, can detect the scents of snakes and other predators and avoid areas where those predators have recently been [1], [2]. In the case of primates, the olfactory sense has been reduced over evolutionary time with concomitant expansion of the visual sense. It has been argued that predation pressure from snakes constituted a strong source of selection that directly favored expansion of the visual system of primates at the expense of the olfactory system [3], [4]. Snakes that had gapes large enough to eat mammals evolved shortly before the primate lineage evolved, and venomous snakes have been a threat to anthropoid primates since their divergence from prosimians approximately 60 million years [4]. Primate visual systems are well-suited for clearly seeing objects in the lower visual field within peripersonal space, where the risk from snakes is usually greatest [4], [5].

Wild primates and both fatal and snake-naïve laboratory-reared primates exhibit strong behavioral responses toward snakes [6]–[10]. Recently, both human and non-human primates have also been found to exhibit strong visual responses toward snakes. In humans, adults, children, and even infants are able to visually detect images of snakes faster than nonthreatening targets both in color and gray-scale images [11]–[15]. Japanese macaques (*Macaca fuscata*) respond in a similar manner [16]. Snake images also receive attentional priority compared to other stimuli. They draw attention away from neutral targets (i.e., a bird) when their images are included against a background of other neutral objects, especially under high perceptual load, i.e., under conditions that simulate the cluttered natural environments in which snakes are often found [17].

A specific neuronal circuit for innate visual recognition of threatening stimuli such as snakes has been proposed that includes a fast, automatic, subcortical pathway involving the superior colliculus and the pulvinar nucleus in the thalamus [3], [4], [18]–[20]. Several behavioral and neurophysiological studies support this model. For instance, monkeys with bilateral neurotoxic lesions of the superior colliculus readily approached food located in a center of a coiled snake model, while monkeys with sham lesions avoided the food [21]. In humans, complete unilateral loss of the pulvinar induced deficits of recognition of emotional expressions while a sparing of the medial and posterior pulvinar did not [22]. When people were shown images of fear faces so quickly that they were not consciously aware of them, both the superior colliculus and pulvinar were activated [23].

In a study to investigate the neuronal basis of the ability of primates to visually detect snakes quickly and automatically, Le et al. [24] found neurons in the medial and dorsolateral pulvinar of Japanese macaques that responded more strongly and quickly to snake images than to images of faces and hands of macaques, and to simple geometric shapes. Interestingly, the response characteristics of these pulvinar neurons were well correlated with those in the superior colliculus except that response latencies were shorter in the superior colliculus [25], [26] as might be expected if the visual route for detecting threatening stimuli goes first to the superior colliculus and then to the pulvinar (see above).

Recently, there has been interest in identifying the specific cues provided by snakes that enable primates to visually detect them so rapidly. Snakes have a number of qualities shared by no other animals, including an elongated, limbless, scaled body that can change shape depending at times on the degree of their intent to strike and thus, on the degree of threat. LoBue and DeLoache [27] found that humans were unable to detect coiled snakes more quickly than other coiled objects. Moreover, elongated snakes were not detected more quickly than flowers. They suggested that the unique ability of snakes to coil upon themselves is the critical stimulus for rapid detection. Unfortunately, they did not control for the level of threat. A study of rhesus macaques (*M. mulatta*) found that a sinusoidal snake model (most similar to an elongated snake) elicited weaker responses than a coiled snake model, as would be expected if a coiled shape is the critical cue. However, a snake model that was partially exposed and revealing only a slight curve elicited stronger responses than a coiled snake model, suggesting that posture per se is less important than the information that can be gleaned from it. A snake poised to strike reveals a greater or more immediate threat than a coiled, sinusoidal, or elongated snake, and indeed, a snake model poised to strike in a threat posture elicited the strongest responses from the monkeys [28]. Children with no experience with snakes also visually detected photos of snakes in threat posture faster than those of snakes not poised to strike [29]. These findings suggest that primates are sensitive to variation in the level of threat revealed by snakes in different positions, with elongated or sinusoidal snakes being least threatening, coiled snakes intermediate, and snakes poised to strike most threatening.

Taking these behavioral and neurological findings together, we predicted that pulvinar neurons would respond preferentially to images of snakes in threatening postures compared to less threatening postures in bottom-up visual processing. To test this prediction, 3 photos of snakes in threat displays with open mouths and 3 photos of snakes in less threatening postures were presented to Japanese macaques in a delayed non-matching to sample (DNMS) task. Here, we report that pulvinar neurons discriminate postural cues of snakes that are linked to their level of threat, consistent with behavioral studies [28], [29].

## Materials and Methods

### Subjects

Two adult (1 female and 1 male) Japanese macaques, weighing 7.0–8.8 kg, were used in this experiment. Each monkey was individually housed in indoor each cage (0.865 m×0.710 m×0.880 m) on a 12-hr on/12-hr off lighting schedule with food available ad libitum. The monkeys were deprived of water in their home cage and received juice as a reward during training and recording sessions. Supplemental water and vegetables were given after each day's session. To assess the monkeys' health, their weight was routinely monitored. The monkeys were treated in strict compliance with the United States Public Health Service Policy on Human Care and Use of Laboratory Animals, the National Institutes of Health Guide for the Care and Use of Laboratory Animals, and the Guidelines for the Care and Use of Laboratory Animals of the University of Toyama. This study was approved by the Committee for Animal Experiments and Ethics at the University of Toyama (Permit Number: A2013MED-46). Environmental enrichment (toys) was provided daily, and all surgery was performed under anesthesia, and all efforts were made to minimize suffering. No monkeys were sacrificed. The animals' health status was monitored throughout the experiment, and kept in a good condition for the animals to perform the task correctly (see below).

### Experimental setup

The monkey sat in a monkey chair 68 cm away from the center of a 19-inch computer display for behavioral tasks during the training and recording sessions in a shielded room. The CRT monitor was set so that its center was on the same horizontal plane as the monkey's eyes. The monkey chair was equipped with a responding button, which was positioned so that the monkey could easily manipulate it. An infrared charge-coupled device (CCD) camera for eye-movement monitoring was firmly attached to the chair by a steel rod. During training and recording sessions, the monkey's eye position was monitored with 33 ms time resolution by an eye-monitoring system [30]. The juice reward was accessible to the monkey through a small spout controlled by an electromagnetic valve. A visual stimulus generator (ViSaGe MKII Visual Stimulus Generator, Cambridge Research Systems, UK) controlled the electromagnetic valve, the timing of visual stimuli onset.

### Visual stimuli

**Figure 1:**
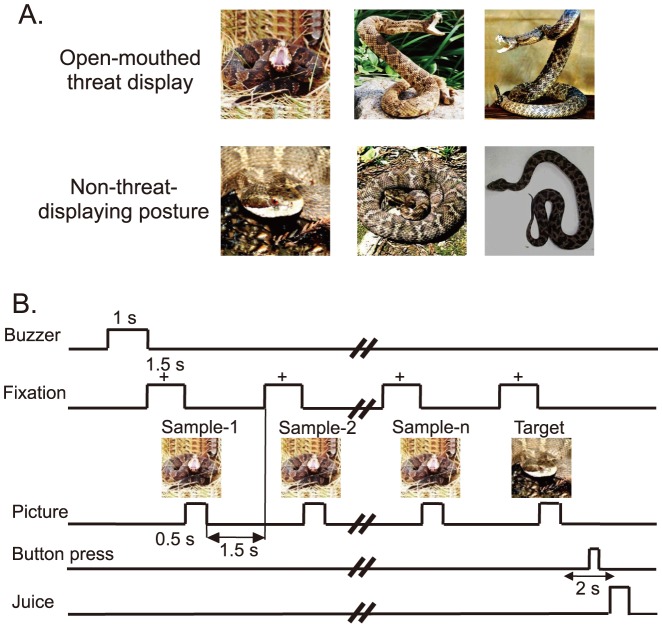
Visual stimuli (A) and delayed nonmatching-to-sample (DMNS) task (B) used in the present study. (A) Six photos of two categories of the stimuli including snakes in threat display and non-threat-displaying postures. (B) Stimulus sequence in the DMNS task in which stimuli were sequentially presented with a delay.

Figure 1A shows the stimulus set, consisting of 3 photos of snakes in open-mouthed, threat display and 3 in closed-mouth, non-threat-displaying postures. All images are of live venomous vipers. We used color images because previous studies reported that color facilitates detection of snakes [13], [31] and we wanted to simulate snakes as closely as they appear in nature. The stimuli were 256 digitized RGB color-scale images with their resolution of 270×270 pixels. Stimuli were presented on a black background of 0.7 cd/m^2^ with their centers at the center of the display. The luminance of each stimulus was determined by measuring luminance of the circular area (radius, 6.35 cm) including each stimulus inside the circle by means of a luminance meter (BM-7A; Topcon, Tokyo). The luminance of these color stimuli was similar (6.005–6.445 cd/m^2^) [luminous intensity (total luminance) ranged from38.432 to 41.248mcd]. These stimuli were displayed on a CRT monitor with a resolution of 640×480 pixels, and the size of the stimulus area was 5–7×5–7°.

Our procedures follow those in Le et al. [24]. In that study, however, pulvinar neurons were not examined for postural differences.

### Behavioral tasks

The monkeys were trained to perform a sequential delayed non-matching-to-sample task (DNMS) that required the discrimination of the visual stimuli (Figure 1A and B) [24]. As illustrated in Figure 1B, the task was initiated by a buzzer tone. Then, a fixation cross was appeared in the center of the display. When the monkeys fixated on the cross for 1.5 s within 0.5–1.0° window, a sample stimulus was presented for 500 ms (sample phase). The control phase was defined as the 100-ms period before the sample phase. Then, after an interval of 1.5 s, the same stimulus appeared again for 500 ms between 1 and 4 times (selected randomly for each trial). Finally, a new stimulus was presented (target phase). When the target appeared, the monkey was required to press a button within 2 s in order to receive a juice reward (0.8 mL). When the monkey failed to respond correctly during the target phase or to press the button before the target phase, the trials were aborted and a 620-Hz buzzer tone was presented. The intertrial intervals (ITI) lasted15–25 s (Figure 1B).

### Training and surgery

The monkeys were trained to perform DNMS task for 3 h/day, 5 day/week. The monkeys reached a 96% correct-response rate after 3 months training. After completion of this training period, a head-restraining device (a U-shaped plate made of epoxy resin) was attached to the skull under aseptic conditions [32], [33]. Subjects were initially anesthetized with a combination of medetomidine hydrochloride (0.5 mg/kg, i.m.) and ketamine hydrochloride (5 mg/kg, i.m.), and anesthesia was maintained by sodium pentobarbital (25 mg/kg, i.m.). For each monkey, the U-shaped plate was anchored with dental acrylic to titanium bolts that were inserted into the skull. We also implanted a reference pin, the location of which was based on the zero coordinates defined in the stereotaxic atlas of the brain of *M. fuscata* individuals [34]. During surgery, heart and respiratory functions and rectal temperature were monitored (LifeScope 14; Nihon Kohden Corporation, Tokyo, Japan). A blanket heater was used to keep body temperature at 36±0.5°C. Antibiotics were administered topically and systemically for 1 week after the surgery in order to prevent infection. Two weeks after surgery, each monkey was retrained while the head was painlessly fixed to the stereotaxic apparatus with the head-restraining device. The performance criterion (>90%) was again attained within 2 weeks.

### Stereotaxic localization of the pulvinar for recording

Before recording from the pulvinar in each hemisphere, a tungsten marker (diameter: 500 µm) was inserted near the target area under anesthesia, and three-dimensional magnetic resonance imaging (3-D MRI) scans of the monkey head were performed. The 3-D pictures of the monkey brain with the marker were reconstructed by computer rendering. Three-dimensional stereotaxic coordinates of the target area were determined in reference to the marker in the 3-D reconstructed brain [35]. Since the recording from the two monkeys is in progress, the two monkeys are alive in the present time, and pulvinar neurons were stereotaxically plotted on the 3-D reconstructed brain [24].

### Electrophysiological procedures and data acquisition

After the monkeys relearned the DNMS task at a rate greater than 85% correct, we commenced daily recording of neuronal activity during the DNMS task. Neuronal activity was recorded from each hemisphere in both subjects. A glass-insulated tungsten microelectrode (0.8–1.5 MΩ at 1 kHz) was stereotaxically inserted into the pulvinar vertically to the orbitomeatal plane in a stepwise fashion by a pulse motor-driven manipulator (SM-21; Narishige Scientific Instrument Lab, Tokyo, Japan). Only neuronal activities with a signal-to-noise ratio greater than 3∶1 were recorded. The analog signals of the neuronal activities, the triggers for visual stimuli, juice rewards, button pressing, and the X-Y coordinates of eye position were digitized at a 40-kHz sampling rate and stored in a computer through a multichannel acquisition processor (MAP; Plexon Inc., Dallas, TX, USA) system. The digitized neuronal activities were isolated into single units by their waveform components using the Offline Sorter program (Plexon Inc.). Superimposed waveforms of the isolated units were drawn in order to assess the variability throughout the recording sessions and then transferred to the NeuroExplorer program (Nex Technologies, Littleton, MA, USA) for further analysis.

### Analysis of basic characteristics of pulvinar neurons

Spike sorting was performed with the off-line sorter program for cluster analysis (Off-line sorter, Plexon Inc.). Each cluster was checked manually in order to ensure that the cluster boundaries were well separated and that the waveform shapes were consistent with the action potentials. For each isolated cluster, an autocorrelogram was constructed, and only units with refractory periods greater than 1.2 ms were used for further analyses. Finally, superimposed waveforms of the isolated units were drawn in order to check the consistency of the waveforms. In addition, all pulvinar neurons were analyzed by autocorrelograms. The autocorrelograms indicated that the refractory periods of the all pulvinar neurons were greater than 2 ms throughout the recording sessions, which indicates that the isolated spikes were recorded from single neurons.

We analyzed single neuronal activity during the following 2 periods: 100 ms before (pre) and 500 ms after (post) the onset of stimulus presentation in the sample phase. The baseline firing-rate was defined as the mean firing rate during the 100-ms pre period. Significant excitatory or inhibitory responses to each stimulus were defined by a Wilcoxon signed-rank (WSR) test (p<0.05 for statistical significance) of the neuronal activity between the 100-ms pre and the 500-ms post periods. In order to investigate the temporal changes in the neuronal responses, the 500-ms post period was further divided into ten 50-ms epochs. The mean neuronal firing rate was calculated for each of these epochs. The response magnitude was defined as follows: the mean firing rate in each epoch minus the mean firing rate during the 100-ms pre period. Each neuron was categorized based on a t-test (P<0.05), in which response magnitudes to all 3 snakes in threat display were compared with those to all 3 snakes in non-threat-displaying postures. For this analysis, two peri-event histograms for the two categories of snakes were constructed using the entire set of data for all trials and all stimuli. “Threat” neurons were defined as such if the response magnitudes to the snakes in threat display were larger than those to the snakes in non-threat-displaying postures (P<0.05). “Non-threat” neurons were defined as such if the response magnitudes to the snakes in non-threat-displaying postures were larger than those to the snakes in threat display (P<0.05). Equal neurons were defined as such if there was no significant difference in response magnitudes between the categories of snakes (P>0.05). Furthermore, in each neuron, mean response magnitudes to the snakes in threat display and snakes in non-threat-displaying postures were computed. Then, grand averaged response magnitudes over all responsive neurons to snakes in threat display and snakes in non-threat-displaying postures were compared by paired t-tests (p<0.05).

In addition, we analyzed the response latency to each visual stimulus. For each neuron, 1 peri-event histogram was constructed with the entire set of data for all trials and all stimuli. Neuronal response latency was defined as the interval from the onset of stimulus presentation to the time at which the neuronal firing rate exceeded the mean ±2 SD of the baseline firing-rate. Averaged response latencies over all responsive neurons to the snakes in threat display and snakes in non-threat-displaying postures were compared by paired t-tests (p<0.05). All data were expressed as mean ± SEM.

### Multidimensional Scaling analysis (MDS)

Multidimensional scaling (MDS) is a method that is used to simplify the analysis of relationships that exist within a complex array of data. MDS constructs a geometric representation of the data in order to show the degree of the relationship between stimuli that are represented by the data matrix (see Young and Hamer [36] for more details). MDS has been used to examine stimulus relationships with data matrices representing neural activity in response to the particular stimulus array (i.e., snakes, monkey and human faces, monkey hands, eye- and face-like patterns, and simple geometrical figures) [24]–[26]. In the present study, the 6 visual stimuli were used to elicit neuronal activity in the pulvinar.

Data matrices of neural activity in a 78×6 array derived from the 82 visually responsive neurons were generated. Euclidean distances as dissimilarity between all possible pairs of 2 visual stimuli were calculated by using the visual responses of the 82 pulvinar neurons. Then, the MDS program (PROXSCAL procedure, SPSS statistical package, version 16) positioned the visual stimuli in the 2-dimensional space with the distances between the stimuli representing the original relationships (i.e., Euclidean distances in the present study) [37], [38]. Finally, the clusters of the visual stimuli were evaluated by discriminant analysis.

## Results

### Characteristic of pulvinar neuronal responses to snakes

**Figure 2:**
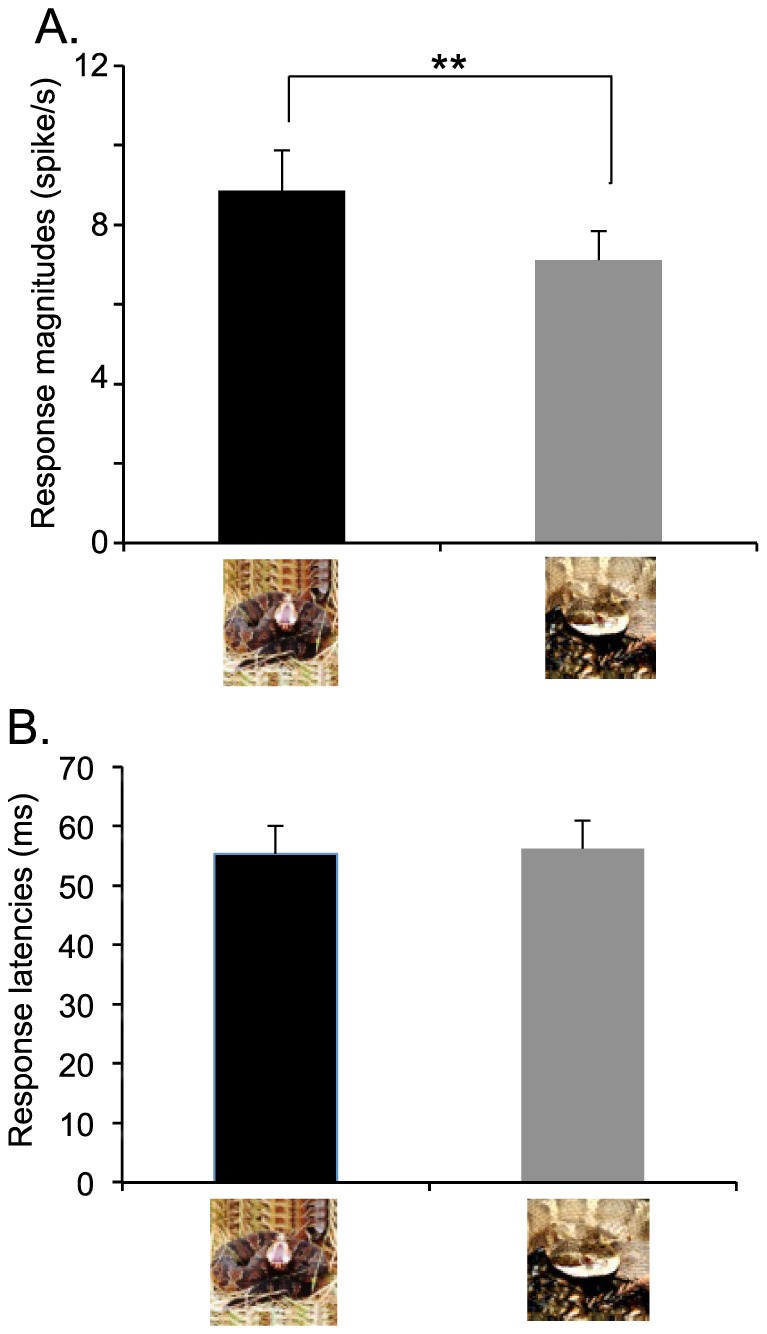
Comparison of mean response magnitudes (n = 78) (A) and latencies (n = 52) (B) to the snakes in threat display vs. non-threat-displaying postures. There was no significant difference between response latency to threatening snakes and snakes in non-threat-displaying postures (paired t-test, t(51) = 0.424, P = 0.673). In contrast, mean response magnitudes to snakes in threat display were significantly larger than to non-threat-displaying snakes. ** significant difference (paired t-test, t(77) = 3.056, P = 0.003). Columns and error bars indicate means with SEM.

Of 821 neurons recorded, 115 neurons responded to the visual stimuli. Of these neurons, 78 neurons were tested with all stimuli in this experiment. From this 78-neuron subset, 45 neurons responded more strongly to snakes in threat display (threat neurons) (t-test, P<0.05), 24 neurons responded more strongly to snakes in non-threat-displaying postures (non-threat neurons) (t-test, P<0.05), and 9 neurons showed no difference between these two kinds of the stimuli (equal neurons) (t-test, P>0.05). Statistical analysis indicated that there was a significant difference in the ratios of these 3 types of neurons [χ^2^-test, χ^2^(2) = 25.0, P = 3.45×10^−6^]. Post-hoc tests indicated that the ratio of the “threat” neurons was significantly greater than that of the “non-threat” neurons (Ryan's method with adjusted significance level, P = 0.016). Furthermore, mean response magnitudes were significantly greater to snakes in threat display than to snakes in non-threat-displaying postures (paired t-test, t(77) = 3.056, P = 0.003) (Fig. 2A). There was no significant difference in response latencies to the snakes between the threat displays and non-threat-displaying postures (55.3±4.7 and 56.2±4.7 ms, respectively, paired t-test, t(51) = 0.424, P = 0.673) (Fig. 2B).

### Multi-dimensional scaling analysis

**Figure 3:**
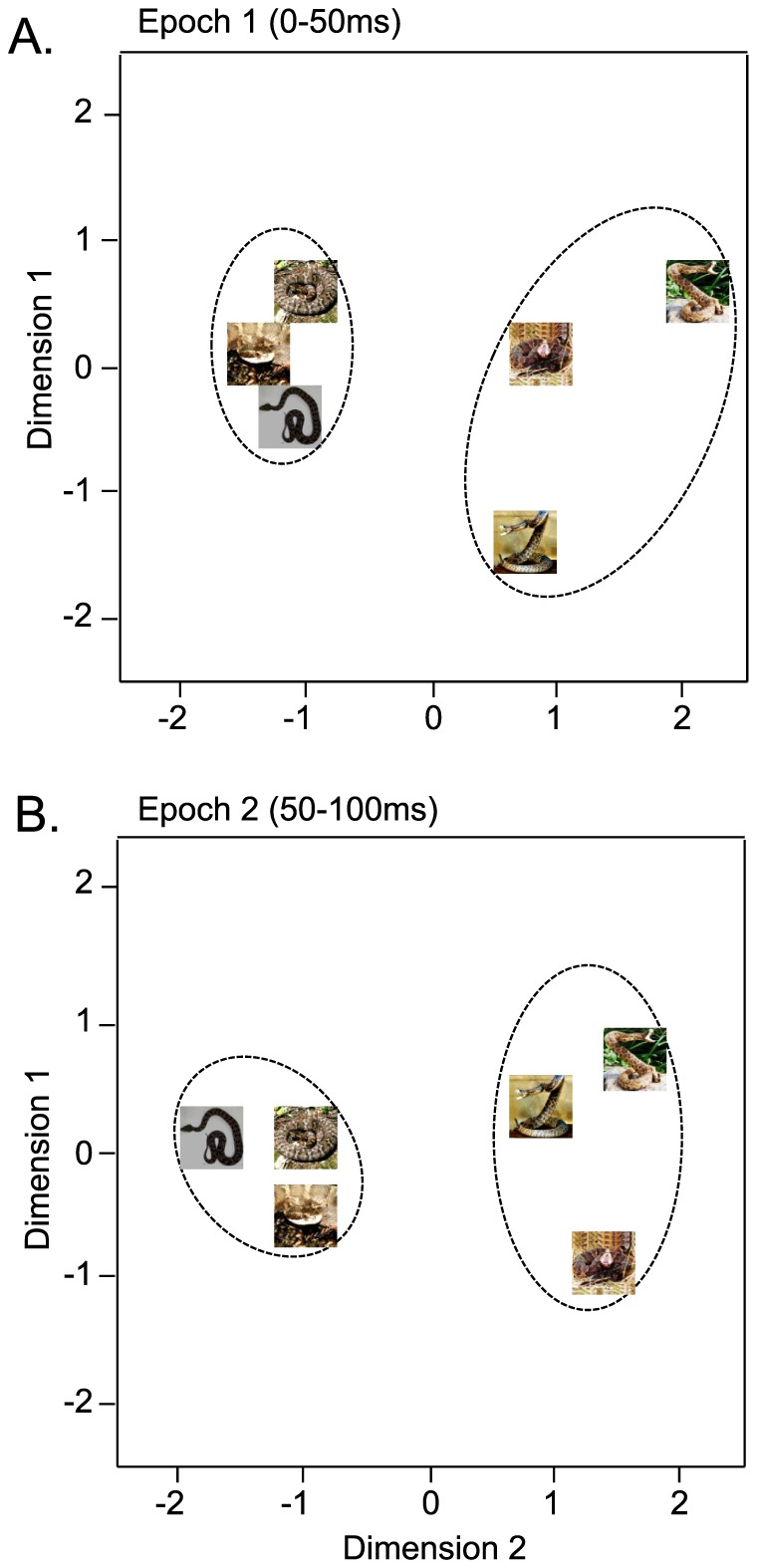
Distributions of the 6 visual stimuli in a two-dimensional space resulting from multidimensional scaling using responses of the 78 neurons to these stimuli in epoch 1 (A), epoch 2 (B). In epochs 1 and 2 (A, B), the snakes in threat display were separated from the non-threat-displaying snakes.

The data sets of response magnitudes of the 78 visually responsive pulvinar neurons in epochs 1 (0–50 ms) and 2 (50–100 ms) after stimulus onset were subjected to multidimensional scaling (MDS) analysis (Fig. 3). After measurement of R^2^ and stress value for up to four dimensions, two-dimensional space showed the best results. In the two-dimensional spaces, R^2^ values of epochs 1 and 2 were 0.94098 and 0.98019, respectively. In both epochs, two clusters, one for snakes in threat displays and another for snakes in non-threat-displaying postures, were recognized. Discriminant analyses showed that correct percent of discrimination was 100% in both epochs (p = 0.01 and p = 0.013, respectively).

### Locations of pulvinar neurons

The pulvinar neurons that responded to the visual stimuli were located at the medial and dorsolateral parts of the pulvinar, consistent with our earlier study [24].

## Discussion

We found here that neurons in the medial and dorsolateral pulvinar responded more strongly to snakes with open-mouthed threat displays compared with snakes with non-threat-displaying postures. In addition, the MDS results indicated that threat-displaying snakes were separated from non-threat-displaying snakes as early as epoch 1. Snakes threatening to strike are more dangerous than those in other postures [39], [40], and monkeys apparently perceive this because they react behaviorally most strongly to snakes poised to strike, less to coiled snakes, and least to sinusoidal snakes [28]. In a previous study, we found that pulvinar neurons responded more strongly and more quickly to snakes compared with faces and hands of monkeys and simple geometrical patterns [24]. Our present results, together with previous research, suggest that the pulvinar plays an important role not only in detecting snakes but also in assessing the degree of threat posed by them. There is an urgency from snakes preparing to strike that requires immediate and focused attention if one is to avoid being bitten. An important function of the pulvinar is in filtering out irrelevant visual stimuli [41]. The greater response magnitudes in pulvinar neurons that we found to snakes poised to strike may assist in focusing the primate's visual attention on the snake.

Response latencies to snakes in general are shorter compared with other stimuli [24], [25], [42]. Nevertheless, here we found no significant differences in response latencies between snakes with and without threat displays. In nature, snakes often rely on ambush to kill their prey. By definition, ambushing requires predators to remain undetected until they strike. Many snakes have evolved coloration and patterns that help to camouflage them, and their narrow, limbless bodies allow them to blend among the background vegetation, reducing their detectability and making it challenging even for primates to see them [4], [43]. If ambush predators are detected and monitored, they lose the element of surprise and are much less dangerous. While it is not necessary to detect snakes from far away, it would be beneficial for their primate prey to detect them before they prepare to strike. Indeed, as snakes can strike quickly, the ability to detect them within striking range regardless of their posture may be crucial for avoiding potentially deadly snakebites. It should be noted, however, that the snake images were presented in the central visual field, where differences in behavioral response latencies among stimuli are less than those in the peripheral visual field in humans [44]. Further studies are required to investigate neuronal responses to snake images presented in the peripheral visual fields.

In summary, this study contributes new neurophysiological evidence that further supports that the pulvinar in primates is highly responsive to snake visual stimuli [24]; population activity of neurons in the medial and dorsolateral pulvinar of Japanese macaques differentiates between snakes presenting a greater threat from those presenting a lesser threat as early as 50 ms after stimulus onset. This fast visual information processing suggests that snake images are processed in a bottom-up visual pathway to the pulvinar. The ability to quickly detect snakes visually and selectively focus on them while also quickly cueing in on their level of threat from their posture would appear to have clear a evolutionary benefit. Our results are thus consistent with the Snake Detection Theory, which argues that snakes provided a novel selective pressure that contributed importantly to the origin of primates and the evolution of the their visual system [3], [4].

## Supporting Information

Checklist S1 ARRIVE (the Animal Research: Reporting of In Vivo Experiments) report.
(DOC)
